# Persistence to extended adjuvant endocrine therapy following Breast Cancer Index (BCI) testing in women with early-stage hormone receptor-positive (HR +) breast cancer

**DOI:** 10.1186/s12885-023-11104-w

**Published:** 2023-06-30

**Authors:** Julia Foldi, Anastasia Tsagianni, Max Salganik, Catherine A. Schnabel, Adam Brufsky, G. J. van Londen, Lajos Pusztai, Tara Sanft

**Affiliations:** 1 Section of Medical Oncology, Yale School of Medicine, New Haven, CT 06510 USA; 2 Present Address: Division of Hematology and Medical Oncology, University of Pittsburgh School of Medicine, Pittsburgh, PA 15213 USA; 3 Biotheranostics Inc, A Hologic Company, San Diego, CA 92121 USA

**Keywords:** Breast Cancer Index, Extended adjuvant endocrine therapy, Endocrine therapy adherence

## Abstract

**Purpose:**

Extending adjuvant endocrine therapy (ET) beyond the standard 5 years offers added protection against late breast cancer recurrences in women with early-stage hormone receptor-positive (HR +) breast cancer. Little is known about treatment persistence to extended ET (EET) and the role that genomic assays may play. In this study, we evaluated persistence to EET in women who had Breast Cancer Index (BCI) testing.

**Methods:**

Women with stage I-III HR + breast cancer who had BCI testing after at least 3.5 years of adjuvant ET and ≥ 7 years of follow-up after diagnosis were included (*n* = 240). Data on medication persistence was based on prescriptions in the electronic health record.

**Results:**

BCI predicted 146 (61%) patients to have low – BCI (H/I)-low – and 94 (39%) patients to have high likelihood of benefit from EET (BCI (H/I)-high). Continuation of ET after BCI occurred in 76 (81%) (H/I)-high and 39 (27%) (H/I)-low patients. Non-persistence rates were 19% in the (H/I)-high and 38% in the (H/I)-low group. The most common reason for non-persistence was intolerable side effects. Patients on EET underwent more DXA bone density scans than those who stopped ET at 5 years (mean 2.09 versus 1.27; *p* < 0.001). At a median follow-up of 10 years from diagnosis, there were 6 metastatic recurrences.

**Conclusions:**

In patients who continued ET after BCI testing, the rates of persistence to EET were high, particularly in patients with predicted high likelihood of benefit from EET. Use of EET is associated with increased use of DXA scans.

**Supplementary Information:**

The online version contains supplementary material available at 10.1186/s12885-023-11104-w.

## Background

Patients with early stage hormone receptor positive (HR +) breast cancers have a risk of distant recurrence that remains high over a prolonged period, with approximately 50% of recurrences occurring more than 5 years after diagnosis despite optimal locoregional and standard adjuvant systemic therapies [1–3]. Several randomized clinical trials established that extending ET beyond the standard 5 years of tamoxifen, an aromatase inhibitor (AI) or a combination of the two, offers superior protection against late recurrences [4–11]. The ideal duration of EET in all-comers remains unclear with some studies suggesting equivalent survival in patients taking 7 or 8 years of total ET compared with 10 years [12, 13], however, certain high risk patients may benefit from longer duration of EET.

The absolute benefit of EET is modest – approximately 3–5% across studies – which must be weighed against added toxicities. An increased number or rare but serious side effects including endometrial cancer and thromboembolic disease were seen in trials of extended tamoxifen. A meta-analysis including seven trials and a total of 16,349 patients analyzed toxicity of EET with AIs [13]: longer treatment was associated with increased rates of cardiovascular events, bone fractures and discontinuation of treatment due to adverse events. In the ABCSG-16/SALSA trial [12] of extended ET, there was an increased risk of bone fractures in the 5-year versus the 2-year group with a hazard ratio (HR) of 1.35 (95% confidence interval [CI], 1.00–1.84).

Studies have shown that early discontinuation of adjuvant ET is associated with increased mortality [14], hence there is growing interest in studying non-compliance, adherence and persistence to ET. Differences between these terms are subtle but they are not interchangeable. Rates of *adherence*, defined as the extent to which patients take medications as prescribed, measured over the first 5 years of adjuvant therapy are estimated to range from 41 to 72% in one systematic review [15], which included data from 29 retrospective studies. In a series of randomized controlled trials of adjuvant ET, early discontinuation rates ranged from 8 to 28% [6, 16–18]. Data on medication taking behaviors beyond 5 years is scarce. *Non-compliance*, defined as early discontinuation of letrozole for any reason, excluding death or breast cancer recurrence, was 18.4% at 2.5 years of EET in the IDEAL trial [19, 20]. One retrospective study examined real-world *persistence,* defined as the time from initiation to discontinuation of treatment, to EET after 5 years in 89 patients who were offered extended therapy based on clinicopathologic risk and found a high rate (78%) of persistence [21].

Breast Cancer Index (BCI) is a multigene-expression based assay that consists of two functional biomarker panels – the Molecular Grade Index (MGI) and the HOXB13/IL17BR ratio (H/I) – that interrogate proliferation and estrogen signaling pathways. BCI has been validated in multiple randomized trial cohorts [22–26] as a prognostic tool for risk of late distant recurrence and predictive of benefit from EET [22–24, 27]. We previously reported on the impact of BCI testing on patient decisions regarding EET [28, 29]. In this study, we aimed to characterize patient experiences and examine persistence rates to EET over a 5-year period after BCI testing at two academic centers in the United States.

## Methods

### Patients

The study was approved by the Institutional Review Boards (IRB) at both Yale University (HIC #200,030,895) and the University of Pittsburgh (HCC 21–197). Given minimal risk to patients, the IRBs determined that the study was exempt from having to obtain informed consent. Eligible patients were identified through review of clinical records of test submission to Biotheranostics (San Diego, CA) and included patients with a history of HR + , stage I-III breast cancer, who had BCI testing sent by their treating oncologist as part of routine care between August 2013 and June 2018, after at least 3.5 but no more than 6 years of adjuvant ET with at least 7 years of total follow-up from breast cancer diagnosis. Patient information including demographic data, clinical and pathological characteristics (TNM stage, tumor grade, ER, PR and HER2 status), treatment history including side effects from ET, the number of dual-energy x-ray absorptiometry (DXA) bone density scans performed from the time BCI was sent until date of last follow-up or when the patients completed adjuvant ET, and information about bone health (normal bone density, osteopenia or osteoporosis) were collected. Data on patient decision regarding continuation of adjuvant ET beyond 5 years was obtained from prescriptions and provider documentation and endocrine therapy persistence was based on valid, continuously active prescriptions in the electronic health record.

### Definition of persistence

We use “persistence” to describe patients’ medication taking behavior after BCI testing. We define persistence as a categorical variable (i.e., “yes” continued/completed EET as planned or “no” did not continue/complete EET as planned). This is a specific aspect of adherence, which is defined as the extent to which patients take medications as prescribed. While different studies define these terms in different ways, we defined non-persistence as an intentional act by patients. Following this logic, patients who discontinued treatment due to either a recurrence of their breast cancer or due to medical advice from their physician were considered persistent. Similarly, patients who died of a cause unrelated to breast cancer and were taking their medication shortly before their death were also considered persistent. The reason for this definition is that non-persistence as an intentional act was the area of interest in this study because it may be a target for interventions. Reasons for discontinuation were characterized for descriptive analysis.

### Molecular testing

BCI testing was done as part of routine clinical care. Briefly, BCI is an assay performed on formalin-fixed paraffin-embedded (FFPE) tissue sections at Biotheranostics (San Diego, CA). The assay combines a proliferation signature known as MGI (Molecular Grade Index) and a two-gene endocrine treatment sensitivity signature based on the ratio of HOXB13 and IL17BR expression (H/I) [30, 31]. The report contains a composite score, based on the combination of the MGI and (H/I) ratio, indicating a percentage risk of late distant recurrence (between 5–10 years post-diagnosis) and a separate categorical (BCI (H/I)-high versus -low) likelihood of benefit from EET. The percent risk of late recurrence is also categorized into low- (BCI risk ≤ 4.9%) versus high-risk (BCI risk > 4.9%) groups. In 2016, Biotheranostics launched a separate prognostic model for patients with 1–3 positive lymph nodes (N1); however, given that most patients in our cohort had BCI testing prior to 2016 and thus were tested using the original BCI model, we used the cutoffs for low- and high-risk groups based on the original model. Of note, our cohort includes nine (4%) patients with 4–9 positive lymph nodes (N2) who at the time were eligible for BCI testing. The BCI assay was performed on archived tumor tissues from patients’ primary breast cancer diagnostic core biopsy or resection specimen and results were reported to the treating physician who discussed them with the patient.

### Statistical analyses

Comparisons between scale variables (e.g., age, number of DXA scans) were made using two-sided independent sample t test and a significance level of *p* < 0.05. Comparisons between nominal parameters were determined using Chi-squared or Fisher’s Exact Test and a significance level of *p* < 0.05.

## Results

### Patient and tumor characteristics

Two hundred forty women were included in our analysis: 198 from Yale University and 42 from University of Pittsburgh. Table 1 summarizes patient and tumor characteristics. Median age was 64 years (range: 41–89); 210 of women (88%) were postmenopausal at the time of BCI testing. 221 patients (92%) had estrogen and progesterone receptor positive and 226 (94%) had human epidermal growth factor receptor 2 (HER2)-negative tumors. 188 patients (78%) had N0, 43 (18%) had N1 and 9 (4%) had N2 disease. 99 patients (41%) received chemotherapy prior to starting adjuvant ET. The majority of patients (*n* = 141, 59%) received an AI as adjuvant ET; 43 (18%) had tamoxifen and 56 (23%) had a combination of tamoxifen and AI. 64 patients (26.5%) had normal bone density, 113 (47%) had osteopenia and 44 (18.5%) had osteoporosis on their most recent DXA scan; 19 patients (8%) had no DXA scans on record.

**Table 1:** Clinicopathologic and treatment characteristics

Characteristic	All patients *N* = 240
**Age**, years	
Median	64
Range	41–89
**Race and ethnicity**, n (%)	
White (non-Hispanic/Latino)	210 (88)
Black/African American	15 (6)
Asian	5 (2)
Hispanic/Latino	5 (2)
Unknown	5 (2)
**Menopausal status**, n (%)	
Pre/Perimenopausal	30 (12.5)
Postmenopausal	210 (87.5)
**Hormone receptor status**, n (%)	
ER + PR +	221 (92)
ER + PR-	18 (7.5)
ER-PR +	1 (0.5)
**HER2 status**, n (%)	
HER2 +	14 (6)
HER2-	226 (94)
**Histologic grade**, n (%)	
Grade 1	78 (32.5)
Grade 2	129 (54)
Grade 3	31 (12.5)
Unknown	2 (1)
**Nodal status,** n (%)	
N0	188 (78)
N1	43 (18)
N2	9 (4)
**Stage**	
Stage I	136 (56.5)
Stage II	92 (38.5)
Stage III	12 (5)
**Chemotherapy**, n (%)	
Yes	99 (41)
No	141 (59)
**Endocrine therapy**, n (%)	
Tamoxifen	43 (18)
AI	141 (59)
Tamoxifen/AI	56 (23)
**Last bone density**, n (%)	
Normal	64 (26.5)
Osteopenia	113 (47)
Osteoporosis	44 (18.5)
Not performed	19 (8)

### BCI results and decision to continue ET

**Fig. 1:**
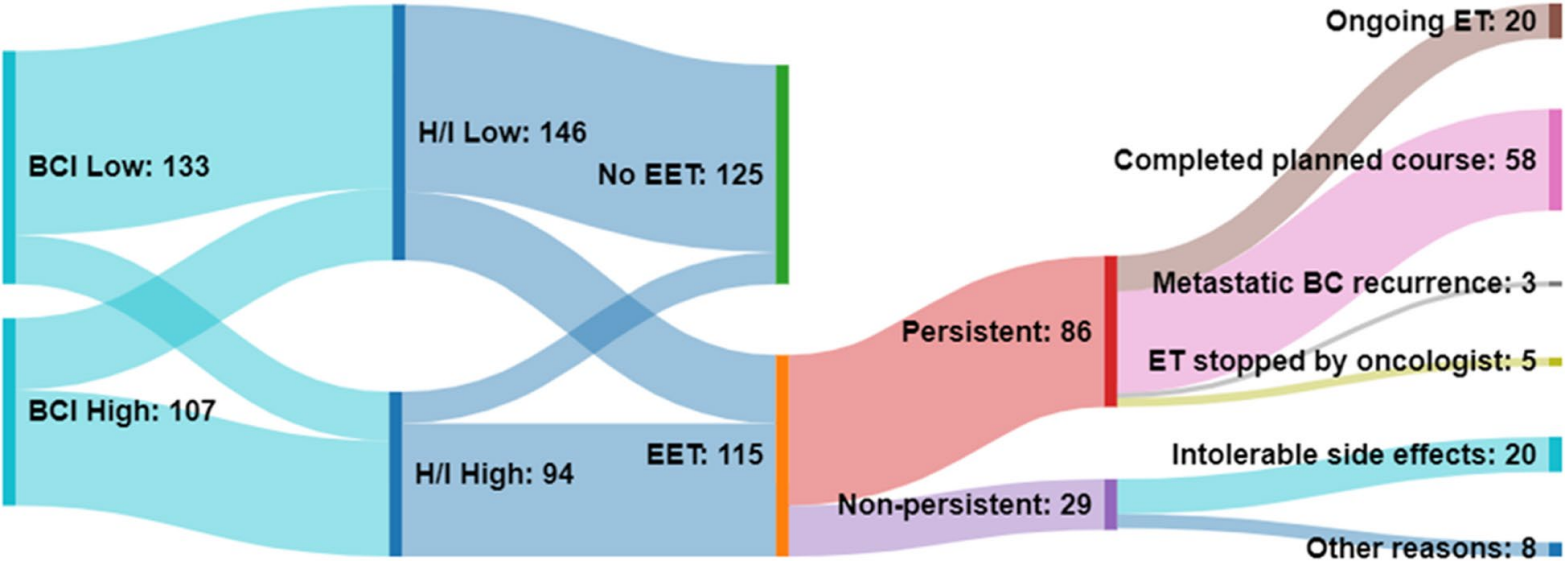
BCI results, patients’ decisions to continue ET and persistence to EET in 240 patients with early-stage HR + breast cancer. Sankey diagram illustrating the relationship between BCI risk of late distant recurrence category (BCI low versus high), likelihood of predicted benefit from extended ET [(H/I)-low versus -high], patients’ decision to stop ET at 5 years (no EET) versus continue beyond 5 years (EET) and persistence to EET and reasons for early discontinuation. The width of the lines is proportional to the patient distribution from one clinical category to the next. Of the 115 patients who continued EET, 86 (75%) remained persistent. Of the 29 patients who were non-persistent, the most common (69%) reason for early discontinuation were intolerable side effects

The overall median risk of distant recurrence based on BCI was 4.6% (range: 1.0%-17.1%): 3.4% (range: 1.0%-4.9%) in the low-risk and 7.8% (range: 5.0%-17.1%) in the high-risk group. BCI categorized 133 patients (55.5%) as being at low- and 107 (44.5%) at high-risk of late.distant recurrence (Table 2 and Fig. 1), while 146 (61%) and 94 (39%) patients were predicted to have a low [(H/I)-low] and high [(H/I)-high] likelihood of benefit from EET, respectively (Table 2 and Fig. 1). 105 (44%) patients were categorized as having both low risk of recurrence and (H/I)-low (low/low) and 66 (27.5%) patients as high risk and (H/I)-high (high/high) (Table 2). The remaining 69 patients were classified into the other 2 possible result categories: 28 were low risk for recurrence but (H/I)-high (low risk/high likelihood of benefit) while 41 were high risk for recurrence but (H/I)-low (high risk/low likelihood of benefit). Patients who continued ET after BCI testing (EET) were younger (*p* = 0.004), had higher grade (*p* < 0.0001), nodal status (*p* = 0.048) and stage (*p* = 0.007) tumors and were more likely to have received chemotherapy (*p* < 0.0001; Supplementary Table 1).

**Table 2:** Summary of Breast Cancer Index (BCI) results

			**Predicted benefit from EET (H/I)**	
			**(H/I)- low**	**(H/I)- high**
**BCI Recurrence Risk Category**	**BCI Recurrence Risk (%; median, range)**	*N* = *240*	*146 (61)*	*94 (39)*
**Low**	3.4 (1.0—4.9)	*133 (55.5)*	105 (44)	28 (11.5)
**High**	7.8 (5.0—17.1)	*107 (44.5)*	41 (17)	66 (27.5)

Overall, 115 (48%) patients elected to continue ET after BCI testing; 76 (66%) of them had (H/I)-high while 39 (34%) had (H/I)-low results (Fig. 1 and Table 3). Of the 94 patients who were (H/I)-high, 76 (81%) elected to continue EET. Of the remaining 18 patients (19%) who stopped ET, half of them did so because of BCI results in above categories (e.g., low risk/high likelihood of benefit) or otherwise low clinicopathologic risk as assessed by their oncologist (Supplementary Fig. 1).

**Table 3:** Persistence and non-persistence among 115 patients who continued extended endocrine therapy after BCI testing

	*N* = 76 ((H/I)-high)	*N* = 39 ((H/I)-low)	*N* = 115 (All patients on EET)
**Persistent**	62 (81%)	24 (62%)	86 (75%)
Ongoing EET	14	6	20
ET completed as planned	42	16	58
ET stopped due to BC recurrence	3	0	3
ET stopped by the physician	3	2	5
**Non-persistent**	14 (19%)	15 (38%)	29 (25%)
ET stopped by patient due to intolerable side effects	10	10	20
ET stopped by patient due to other reasons	4	5	9

*Abbreviations*: *BC* Breast cancer, *ET* Endocrine therapy, *EET* Extended endocrine therapy

### EET persistence

Table 3 and Fig. 1 summarize persistence of the 118 patients who continued EET after BCI testing. Overall, 86 (75%) remained persistent: 20 patients were on EET at the time of last follow-up; 58 patients completed EET as planned; 3 patients developed metastatic breast cancer; and 5 patients were advised by their oncologist to discontinue EET due to either non-breast cancer serious illness limiting life expectancy or because of new data suggesting shorter optimal duration of EET. Among the 29 (25%) patients who were non-persistent, the most common (69%) reason for discontinuation was therapy-related side effects.

Of the 115 patients who continued EET after BCI testing, 76 patients were BCI (H/I)-high versus 39 patients who were BCI (H/I)-low. A higher percentage (81%) of patients in the (H/I)-high group remained persistent when compared to the (H/I)-low group (62%) (Table 3) (*p* = 0.0243, Fisher’s Exact Test).

### Long-term outcomes and bone health

Two hundred thirty-one (96%) patients were alive at a median follow-up of 10 years. There were 6 metastatic breast cancer recurrences and 4 of these patients died of their disease. There were 5 additional non-breast cancer related deaths during the follow-up period. 14 (6%) patients developed second primary cancers including 4 contralateral breast cancers (summarized in Supplementary Table 2). Supplementary Table 3 summarizes BCI results, decision to continue ET, total number of years on ET and the number of years from diagnosis to recurrence for the 6 patients who experienced metastatic breast cancer recurrence. Four (67%) were characterized as high-risk by BCI including the numerically highest predicted percent risk of late recurrence (17.1%) while two patients were low risk. Three of them were BCI (H/I)-low and three were BCI (H/I)-high. Four patients experienced recurrences in years 5–6 from diagnosis, in close proximity to BCI testing, while the remaining two patients recurred after 10 years – both were BCI (H/I)-low and elected to stop ET at 5 years.

Table 4 shows the number of DXA scans performed and bone density status on the last scan in patients who continued EET versus those who stopped at 5 years. Women on EET had a mean 2.09 DXA scans performed from the date of BCI testing until last follow-up, compared with 1.27 in women who stopped ET at 5 years (*p* < 0.001). While there were numerically higher numbers of women with osteoporosis in the EET group, the distribution of bone health diagnoses was not statistically significantly different between the two groups (*p* = 0.427; Chi-squared test). Notably, more women in the no EET group (14% vs. 2%) had no DXA scans on record.

**Table 4:** Summary of bone health and bone density scans

			**Number of DXA scans since BCI**	**Bone density status on most recent DXA scan**			
		*N* = *240*	Mean*	**Normal**	**Osteopenia**	**Osteoporosis**	**Not performed**
**ET > 5 years**	**Yes**	115 (48)	*2.09*	35 (29)	53 (46)	25 (23)	2 (2)
	**No**	125 (52)	*1.27*	29 (24)	60 (48)	19 (15)	17 (13)

^*^p < 0.001

## Discussion

This study provides data from long-term follow-up on our previously reported prospective assessment of the decision-making impact of the BCI assay in routine clinical practice in patients who face the important and difficult decision to continue ET or stop after completing 5 years [28]. This is the first study to also assess EET persistence in this context. The results show that patients who elect to continue ET based on BCI testing have a high likelihood of EET persistence: the observed rate of persistence at 75% is similar to a previously reported rate of 71.9% in what is, to the best of our knowledge, the only other study assessing EET persistence in a real-world setting [21]. Perhaps even more impactful is the finding that those patients for whom BCI predicted a high likelihood of benefit from EET had even higher persistence rate to EET at 81%; the difference in persistence rates between the (H/I)-high and -low groups was statistically significant (*p* = 0.0243). This study confirms our prior findings in a larger cohort demonstrating that the majority of BCI (H/I)-high patients (81%) chose to extend ET after BCI testing and discussion with their oncologist. However, BCI test results indicating a low prognostic risk of recurrence and high likelihood of benefit from EET could increase non-compliance with treatment recommendations, i.e., the decision to stop ET despite continued treatment benefit, which we observed in our study.

While the ideal duration of adjuvant ET in all-comers with early-stage HR + breast cancer remains unclear, there is ample evidence for preferential benefit from EET in patients with high BCI (H/I) ratio. (H/I)-high tumor biology corresponds to an approximately 58–67% relative risk reduction from EET in three large cohorts. In MA.17 (*n* = 249), (H/I)-high status was associated with a decrease in late recurrences (odds ratio [OR)] = 0.35; 95% CI, 0.15–0.73; *p* = 0.007) and with an absolute recurrence risk reduction of 16.5% at 5 years [23]. Similarly, in Trans-aTTom (*n* = 789), those patients classified as BCI (H/I)-high derived a significant benefit from 10 versus 5 years of tamoxifen (HR 0.33; 95% CI, 0.14–0.75; 9.7% absolute risk reduction; *p* = 0.016) [32]. Additionally, in 908 patients in the IDEAL trial, high BCI (H/I) significantly predicted benefit from 5 versus 2.5 years of extended letrozole in the overall cohort (HR 0.42; 95% CI, 0.21–0.84; *p* = 0.011) and in the subset of patients who received any AI during the first 5 years of ET (HR 0.34; 95% CI, 0.16–0.73; *p* = 0.004) [27].

In order to achieve the risk reductions above, patients must take their medication as close as possible to as prescribed; available evidence points to early discontinuation of adjuvant ET being associated with increased mortality [14]. Most of the data on adherence and persistence comes from studies done in the first 5 years of adjuvant ET. In a systematic review including data from 29 studies [15], non-adherence over greater than 4 years of therapy with adjuvant ET ranged from 28 to 59%, while non-persistence after 5 years of treatment ranged from 31 to 73%. Our results, and those of Myrick et al. [21] suggest higher persistence rates to EET after 5 years of adjuvant therapy. Since our study only included patients who had BCI testing, our cohort is enriched for patients who were likely persistent and adherent to ET during the initial 5 years and thus, is limited to studying this particular population that is possibly more likely to be adherent and persistent over longer follow-up. However, our results of higher persistence in those with BCI (H/I)-high results suggests the test result itself may be a tool to improve the behaviors associated with medication-taking behaviors.

There has been much interest in various strategies to improve adherence and persistence to adjuvant ET. Most early efforts focused on one-time or short-term educational interventions that emphasized the benefits of treatment; however, these studies demonstrated no improvement in adherence [33, 34]. More recently, mobile health has emerged as a strategy to improve medication adherence in multiple disease settings. For example, the SWOG S1105 randomized trial aimed to use one-way text messaging to increase adherence and persistence to adjuvant AIs and used urine metabolite assays to monitor adherence [34]. Observed adherence rates at three years, irrespective of prior adherence status, were similar in both the text messaging and no text messaging arms at around 55%. Perhaps most strikingly, by year three, 81.9% of patients in the text messaging and 85.6% in the no text messaging arms had an adherence failure event (a urine AI metabolite under a specified level or no submitted specimen). Overall, this study showed high rates of non-adherence to ET within the first 5 years and did not show any improvement with one-way text messaging reminders. There are currently efforts to use bi-directional text messaging that also tracks adherence and collects patient-reported information on side effects and other barriers to taking the medication, which can trigger alerts and interventions [35, 36].

In this study, we show that patients who had prognostic and, in particular, predictive information, from BCI testing had high rates of persistence to EET. Whether availability of BCI results improves persistence is unclear as our study only included patients who were persistent after the initial 5 years of therapy and were motivated to potentially continue ET. Based on our prior results that among patients recommended for EET, 82% reported they were more likely to comply with treatment based on BCI results [29] and with new data from this study, we hypothesize that BCI testing may help motivated patients remain persistent with treatment. This raises the question of whether BCI testing could be incorporated into the armamentarium of strategies improving adherence and persistence earlier during therapy to create an individualized, risk-stratified, treatment plan. Like individualized care plans in the survivorship setting, adjuvant care plans may give patients a roadmap to their adjuvant treatment over 5–10 years. For example, most patients with early-stage HR + breast cancers fall into the BCI low risk and BCI (H/I)-low category and for them, adjuvant care plans would emphasize the goal of 5 years of ET and follow-up with focus on strategies to promote adherence and persistence over those 5 years. Upon completion, they could “graduate” from traditional oncology follow-up to a survivorship program. On the other hand, for patients with BCI high risk and BCI (H/I)-high results, individualized treatment plans would include 10 years of ET and they may require more frequent follow-up or additional strategies to mitigate side effects such as more aggressive management of bone loss or cardiovascular risk factors for those on AIs. Treatment plans for patients with equivocal, i.e., Prognostic High/Predictive Low or Prognostic Low/Predictive High BCI results would be less clear, although BCI (H/I) status has been shown consistently to predict benefit from EET. Whether adjuvant treatment plans incorporating information from BCI testing early in the course of therapy would improve adherence/persistence or lead to reduced anxiety and improved quality of life will need to be tested in future research.

After 10 years of follow-up from diagnosis, there were six patients who experienced distant breast cancer recurrence, consistent with the overall excellent outcome of patients with early-stage HR + breast cancers. Given the small number of recurrences, no conclusions can be drawn regarding the association of BCI results and EET persistence with long-term outcomes. Overall, women who elected to extend ET had more bone density scans performed compared with those who stopped ET at around 5 years, and had numerically higher proportion of patients with osteoporosis, which can lead to increased health care cost. We previously reported on BCI-related projected net savings that were estimated to be US $5,190/patient [29], primarily driven by targeted use of EET.

Our study has several limitations. First, all patients received BCI testing and thus, it represents a population more likely to be persistent with ET in the first 5 years of therapy who are possibly more motivated to continue being persistent. Second, our cohort is small, yet it does include patients from two U.S. institutions servicing a diverse demographic region, which increases generalizability. Third, information on persistence was obtained from prescriptions entered into the electronic health record and from provider notes. In the future, studies incorporating urine metabolite testing to assess adherence to ET after BCI testing should be undertaken.

In summary, our multi-institutional study found high rates of persistence to EET after BCI testing, particularly in patients with (H/I)-high results, which is the population most likely to benefit from 10 years of ET. BCI is commercially available in the United States for all early-stage HR + breast cancer patients with up to 3 positive lymph nodes and is included in the National Comprehensive Cancer Network (NCCN) and American Society for Clinical Oncology (ASCO) guidelines. It is covered by Medicare and many private payors. It is also available in select international locations. The test is typically offered after 5 years of primary adjuvant therapy to help physicians and patients make informed decisions on the benefit of extending endocrine therapy to 10 years. Additionally, there is an ongoing large, population-based prospective BCI Registry study to evaluate long-term clinical outcome, clinical impact, medication adherence and quality of life in patients with HR + early-stage breast cancer receiving BCI testing as part of routine clinical care to inform extended endocrine therapy. Adjuvant, risk-stratified treatment care plans incorporating BCI testing earlier during treatment, along with other strategies, may increase the likelihood of persistence and adherence to ET, which has the potential to improve long-term outcomes in patients with early-stage HR + breast cancers.

## Conclusions

In patients with early-stage HR + breast cancer who received BCI testing after approximately 5 years of adjuvant ET, rates of persistence to extended ET were high, particularly in patients with predicted high likelihood of benefit from EET. BCI can be a clinically valuable tool, in addition to clinicopathologic information, in guiding discussions with patients and decision-making regarding extending adjuvant endocrine therapy.

## Supplementary Information

**Additional file 1: Supplementary table 1.** Patient and tumor characteristics by patients’ decision to continue EET.

**Additional file 2: Supplementary table 2.** Long-term outcomes of all patients.

**Additional file 3: Supplementary table 3.** Distant breast cancer recurrences (*n*=6).

**Additional file 4: Supplementary figure 1. **Sankey diagram illustrating the reasons for discontinuing ET in 94 patients with high likelihood of benefit from EET [(H/I)-high].

## Data Availability

The datasets generated during and analyzed during the current study are not publicly available due to protected patient information but deidentified datasets are available from the corresponding author upon reasonable request.

## Abbreviations

BCI: Breast Cancer Index
HR +: Hormone receptor-positive
ET: Endocrine therapy
EET: Extended endocrine therapy
AI: Aromatase inhibitor
CI: Confidence interval
MGI: Molecular Grade Index
H/I: HOXB13/IL17BR ratio
DXA: Dual-energy x-ray absorptiometry
FFPE: Formalin-fixed paraffin-embedded
HER2: Human epidermal growth factor receptor 2
ER: Estrogen receptor
PR: Progesterone receptor
NCCN: National Comprehensive Cancer Network
ASCO: American Society for Clinical Oncology
