# Supplementary material for: Persistence to extended adjuvant endocrine therapy following Breast Cancer Index (BCI) testing in women with early-stage hormone receptor-positive (HR +) breast cancer
Source: BMC Cancer. 2023 Jun 30;23:606. doi: 10.1186/s12885-023-11104-w (PMC10314405; doi:10.1186/s12885-023-11104-w)
Supplement: Supplementary file 1 — Additional file 1: Supplementary table 1. Patient and tumor characteristics by patients’ decision to continue EET. [file 12885_2023_11104_MOESM1_ESM.docx]

| **Supplementary Table 1. Patient and tumor characteristics by patients’ decision to continue EET** | | | |
| --- | --- | --- | --- |
| **Characteristic** | **EET**  **N=115** | **No EET**  **N=125** | **P-value** |
| **Age**, years |  |  | ***0.004** |
| Median | 62 | 65 |  |
| Range | 41-80 | 45-89 |  |
| **Race and ethnicity**, n (%) |  |  | 0.948 |
| White (non-Hispanic/Latino) | 100 (87) | 110 (88) |  |
| Black/African American | 7 (6) | 8 (6.5) |  |
| Asian | 3 (2.5) | 2 (1.5) |  |
| Hispanic/Latino | 2 (2) | 3 (2.5) |  |
| Unknown | 3 (2.5) | 2 (1.5) |  |
| **Menopausal status**, n (%) |  |  | 0.807 |
| Pre/Perimenopausal | 15 (13) | 15 (12) |  |
| Postmenopausal | 100 (87) | 110 (88) |  |
| **Hormone receptor status**, n (%) |  |  | 0.737 |
| ER+PR+ | 107 (93) | 114 (91) |  |
| ER+PR- | 7 (6) | 11 (9) |  |
| ER-PR+ | 1 (1) | 0 |  |
| **HER2 status**, n (%) |  |  | ***0.018** |
| HER2+ | 11 (9.5) | 3 (2.5) |  |
| HER2- | 104 (90.5) | 122 (97.5) |  |
| **Histologic grade**, n (%) |  |  | ***<0.0001** |
| Grade 1 | 22 (19) | 56 (45) |  |
| Grade 2 | 72 (62.5) | 57 (45.5) |  |
| Grade 3 | 20 (17.5) | 11 (8.5) |  |
| Unknown | 1 (1) | 1 (1) |  |
| **Nodal status,** n (%) |  |  |  |
| N0 | 83 (72) | 105 (84) | ***0.048** |
| N1 | 25 (22) | 18 (14.5) |  |
| N2 | 7 (6) | 2 (1.5) |  |
| **Stage** |  |  | ***0.007** |
| Stage I | 54 (47) | 82 (65.6) |  |
| Stage II | 52 (45) | 40 (32) |  |
| Stage III | 9 (8) | 3 (2.5) |  |
| **Chemotherapy**, n (%) |  |  | ***<0.0001** |
| Yes | 64 (55.6) | 35 (28) |  |
| No | 51 (44.5) | 90 (72) |  |
| **Endocrine therapy**, n (%) |  |  | 0.615 |
| Tamoxifen | 18 (15.5) | 25 (20) |  |
| AI | 68 (59) | 73 (58.5) |  |
| Tamoxifen/AI | 29 (25.5) | 27 (21.5) |  |
| **Last bone density**, n (%) |  |  | 0.427 |
| Normal | 35 (30) | 29 (23) |  |
| Osteopenia | 53 (46) | 60 (48) |  |
| Osteoporosis | 25 (22) | 19 (15) |  |
| Not performed | 2 (2) | 17 (14) |  |
