# Supplementary material for: Persistence to extended adjuvant endocrine therapy following Breast Cancer Index (BCI) testing in women with early-stage hormone receptor-positive (HR +) breast cancer
Source: BMC Cancer. 2023 Jun 30;23:606. doi: 10.1186/s12885-023-11104-w (PMC10314405; doi:10.1186/s12885-023-11104-w)
Supplement: Supplementary file 2 — Additional file 2: Supplementary table 2. Long-term outcomes of all patients. [file 12885_2023_11104_MOESM2_ESM.docx]

| **Supplementary Table 2. Long-term outcomes of all patients** | | |
| --- | --- | --- |
| **Status at last follow-up** | **N=240** | **Details** |
| Alive | 231 |  |
| Deceased | 9 | breast cancer recurrence (4), other causes (5) |
| Alive and event-free | 215 |  |
| Metastatic breast cancer recurrence | 6 |  |
| Local breast cancer recurrence | 0 |  |
| Second primary cancer (includes second breast cancer) | 14 | contralateral primary breast cancer (4) |
| Deceased due to non-breast cancer cause | 5 | COVID-19 (1), STEMI (1), CVA (1), decompensated liver failure (1), bowel perforation (1) |
| Abbreviations: COVID-19: coronavirus disease 2019; STEMI: ST elevation myocardial infarction; CVA: cerebrovascular event | | |
