# Supplementary material for: Persistence to extended adjuvant endocrine therapy following Breast Cancer Index (BCI) testing in women with early-stage hormone receptor-positive (HR +) breast cancer
Source: BMC Cancer. 2023 Jun 30;23:606. doi: 10.1186/s12885-023-11104-w (PMC10314405; doi:10.1186/s12885-023-11104-w)
Supplement: Supplementary file 3 — Additional file 3: Supplementary table 3. Distant breast cancer recurrences (n=6). [file 12885_2023_11104_MOESM3_ESM.docx]

| **Supplementary Table 3. Distant breast cancer recurrences (n=6)** | | | | | |
| --- | --- | --- | --- | --- | --- |
| **BCI Recurrence Risk (%)** | **BCI Risk Category** | **BCI H/I** | **Continued ET after BCI** | **Years on ET before recurrence** | **Years from diagnosis to recurrence** |
| 17.1 | High | High | Yes | 6 | 6 |
| 10.0 | High | High | Yes | 5 | 5 |
| 7.1 | High | Low | No | 5 | 6 |
| 5.6 | High | High | Yes | 5 | 5 |
| 4.9 | Low | Low | No | 5 | 10 |
| 3.8 | Low | Low | No | 6 | 10 |
