# Supplementary material for: Persistence to extended adjuvant endocrine therapy following Breast Cancer Index (BCI) testing in women with early-stage hormone receptor-positive (HR +) breast cancer
Source: BMC Cancer. 2023 Jun 30;23:606. doi: 10.1186/s12885-023-11104-w (PMC10314405; doi:10.1186/s12885-023-11104-w)
Supplement: Supplementary file 4 — Additional file 4: Supplementary figure 1. Sankey diagram illustrating the reasons for discontinuing ET in 94 patients with high likelihood of benefit from EET [(H/I)-high]. [file 12885_2023_11104_MOESM4_ESM.docx]

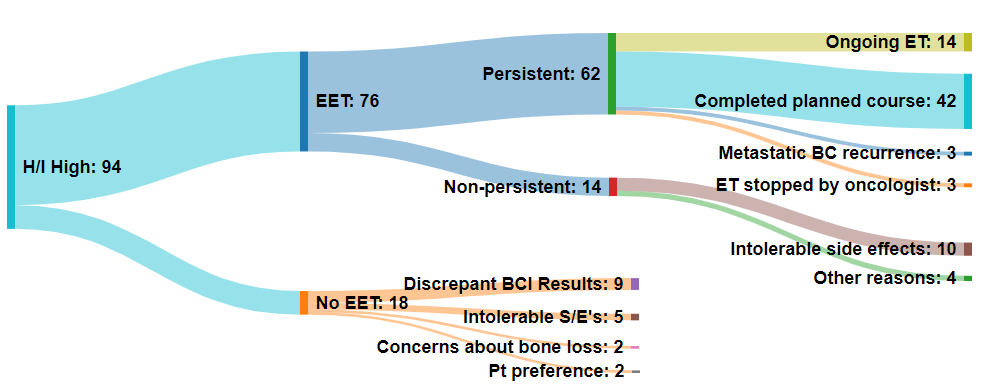


**Supplementary Figure 1.**

Sankey diagram illustrating the reasons for discontinuing ET in 94 patients with high likelihood of benefit from EET (H/I high). The width of the lines is proportional to the patient distribution from one clinical category to the next. Of the 94 H/I high patients, 76 (81%) elected for EET and 18 (19%) stopped ET at around 5 years. Among the 18 patients who stopped ET, the most common reason for stopping ET were equivocal BCI results. Of the 76 patients who continued EET, 62 (81.5%) remained persistent. Among the 14 patients with non-persistence, the most common (71%) reason for early discontinuation were intolerable side effects.
